# Emergent Dynamics and Spatio Temporal Patterns on Multiplex Neuronal Networks

**DOI:** 10.3389/fncom.2021.774969

**Published:** 2021-12-02

**Authors:** Umesh Kumar Verma, G. Ambika

**Affiliations:** Department of Physics, Indian Institute of Science Education and Research Tirupati, Tirupati, India

**Keywords:** multiplex network, neuronal network, synchronization, multi-cluster synchronization, mixed-mode oscillations

## Abstract

We present a study on the emergence of a variety of spatio temporal patterns among neurons that are connected in a multiplex framework, with neurons on two layers with different functional couplings. With the Hindmarsh-Rose model for the dynamics of single neurons, we analyze the possible patterns of dynamics in each layer separately and report emergent patterns of activity like in-phase synchronized oscillations and amplitude death (AD) for excitatory coupling and anti-phase mixed-mode oscillations (MMO) in multi-clusters with phase regularities when the connections are inhibitory. When they are multiplexed, with neurons of one layer coupled with excitatory synaptic coupling and neurons of the other layer coupled with inhibitory synaptic coupling, we observe the transfer or selection of interesting patterns of collective behavior between the layers. While the revival of oscillations occurs in the layer with excitatory coupling, the transition from anti-phase to in-phase and vice versa is observed in the other layer with inhibitory synaptic coupling. We also discuss how the selection of these spatio temporal patterns can be controlled by tuning the intralayer or interlayer coupling strengths or increasing the range of non-local coupling. With one layer having electrical coupling while the other synaptic coupling of excitatory(inhibitory)type, we find in-phase(anti-phase) synchronized patterns of activity among neurons in both layers.

## 1. Introduction

The complexity underlying the patterns of dynamical behavior in the brain is a fascinating and challenging research area in recent times (Sporns, 2013). The complexity arises not only from a large number of neurons involved but also from the variety and plasticity of connections or interactions among them during any type of neuronal or cognitive activity (Pereda, 2014; Ashwin et al., 2016). The interactions can be electrical via gap junction and excitatory or inhibitory interaction via chemical synapses. The collective behavior or synchronization among a large number of neurons is essential for various neurobiological processes, which mostly appear due to the inter neuronal synaptic interactions (Pikovsky et al., 2001). Also, various brain disorders, such as Alzheimer's disease, schizophrenia, Parkinson's disease, and epilepsy, have been linked to the abnormal patterns of synchronization among the neurons (Uhlhaas and Singer, 2006; Jalili et al., 2007; Knyazeva et al., 2010). The nature of the collective dynamics can have different forms of oscillatory patterns that include in-phase oscillations, anti-phase oscillations, multi-cluster oscillations, etc. (Jalan and Singh, 2016; Pournaki et al., 2019). In addition, coupled neurons also show quiescent states due to suppression of activity or amplitude death (AD) (Saxena et al., 2021).

We find the multiplex framework is ideal for describing the collective dynamics of neurons since an assembly of neurons can have excitatory or inhibitory types of electrical or chemical synaptic interactions (Boccaletti et al., 2014; Verma and Ambika, 2021). Then, analysis can be done with the same set of neurons distributed in different layers, based on the nature of interactions among them. In the present study, we consider the framework of multiplex networks to study the activity patterns that can emerge or get selected when neurons in one layer interact with each other through excitatory synaptic couplings and neurons in the other layer interact with each other through inhibitory synaptic couplings. Equally interesting and realistic is the case where one layer of neurons interact electrically while in the other layer, the interaction is synaptic or chemical of excitatory or inhibitory type. We begin by studying the patterns of collective dynamics in each layer separately and observe how excitatory synaptic coupling induces completely synchronized oscillations and AD, while inhibitory synaptic coupling induces anti-phase synchronized oscillations for local connections and multi-cluster oscillations with relative phase ordering for non-local connections. In this context, we note that anti-phase synchronization is observed in neuronal networks in human and animal brains (de la Iglesia et al., 2000; Ueda et al., 2002; Ohta et al., 2005), climactic networks (Hinnov et al., 2002; Saenko et al., 2002), food web (Vandermeer, 2004), and lasers (Wiesenfeld et al., 1990). We note in multiplex neuronal networks with attractive and repulsive interactions, anti-phase synchronization is reported recently (Chowdhury et al., 2021) and chimera states are found to occur in multilayer networks of neurons (Majhi et al., 2016, 2017, 2019).

When both layers are multiplexed, we find transfer or selection of activity patterns across the layers, with the revival of oscillations from AD state in the first layer and a transition from anti-phase to in-phase in the second layer. Depending on the strength of intralayer coupling, activity patterns corresponding to the stronger interaction get selected and stabilized across the neurons in both layers. When one layer has electrical coupling and the other layer with synaptic coupling, in-phase or anti-phase oscillations are induced depending on whether synaptic coupling is excitatory or inhibitory. These activity patterns have rhythmic dynamics with mixed-mode oscillations (MMO), which are complex periodic forms of activity. We note such MMOs are experimentally observed and analyzed in neurophysiological studies (Del Negro et al., 2002; Desroches et al., 2013; Ghosh et al., 2020). We study the transitions between such patterns of activity and how the relevant parameters can be tuned for a specific pattern to get selected across the layers.

## 2. Multiplex Neuronal Networks

We consider a multiplex network of neurons with two layers, each of them consisting of an ensemble of *N* Hindmarsh-Rose (HR) neurons coupled on a regular ring network. We take the neurons in the first layer (L1) to be interacting with each other with excitatory synaptic coupling and those in the second layer (L2) interacting through an inhibitory synaptic coupling. The neurons in L1 interact with neurons in L2 with multiplex like *i* to *i* coupling via feedback. The dynamics of the multiplex network of neurons is thus modeled as shown in Equation (1)


(1) $$\begin{array}{l} {\dot{x}}_{i,1}=B_{i,1}+\frac{\lambda_{1}}{2p_{1}}(V_{s}-x_{i,1})\sum_{k=i-p_{1}}^{i+p_{1}}\Gamma (x_{k,1})+\epsilon x_{i,2}\\ {\dot{y}}_{i,1}=(a+\alpha )x_{i,1}^{2}-y_{i,1}\\ {\dot{z}}_{i,1}=c(bx_{i,1}-z_{i,1}+e)\\ {\dot{x}}_{i,2}=B_{i,2}-\frac{\lambda_{2}}{2p_{2}}(V_{s}-x_{i,2})\sum_{k=i-p_{2}}^{i+p_{2}}\Gamma (x_{k,2})+\epsilon x_{i,1}\\ {\dot{y}}_{i,2}=(a+\alpha )x_{i,2}^{2}-y_{i,2}\\ {\dot{z}}_{i,2}=c(bx_{i,2}-z_{i,2}+e) \end{array}$$

where $B_{i,j}=ax_{i,j}^{2}-x_{i,j}^{3}-y_{i,j}-z_{i,j}$, *i* = 1, 2, …, *N* and *j* = 1, 2 (Majhi et al., 2016). The variable *x*_*i,j*_ represents the action potential, and the variables *y*_*i,j*_ and *z*_*i,j*_ represent the transport of ions across the membrane through fast and slow channels, respectively. The function Γ(*x*_*i,j*_) = 1/{1 + exp[−β(*x*_*i,j*_ − ϕ_*s*_)]} is the sigmoidal chemical synaptic coupling function with *V*_*s*_ as reversal potential. Here, we take the reversal potential *V*_*s*_ = 2 such that *V*_*s*_ > *x*_*i*_(*t*) can be satisfied. We choose the synaptic threshold ϕ_*s*_ = −0.25 and β = 10 in the sigmoidal function. Also, *p*_1_ and *p*_2_ take care of the range of interactions, whether it is local or non-local, with *p*_1,2_ = 1 being local. The other system parameters are *a* = 2.8, α = 1.6, *b* = 9, *c* = 0.001, and *e* = 5 such that the individual HR neurons show regular square-wave bursting dynamics. In the present work, the emergent dynamics of Hindmarsh-Rose (HR)neurons are studied by solving Equation (1), using fourth-order Runge-Kutta method, with initial conditions are chosen randomly between −1 and 1, for various cases as presented below.

### 2.1. Spatio Temporal Patterns on a Single Layer

We begin by considering the emergent dynamics or patterns of activity that can develop in each layer in the absence of multiplexing with ϵ = 0 and number of neurons *N* = 50 in Equation (1).

**Figure 1:**
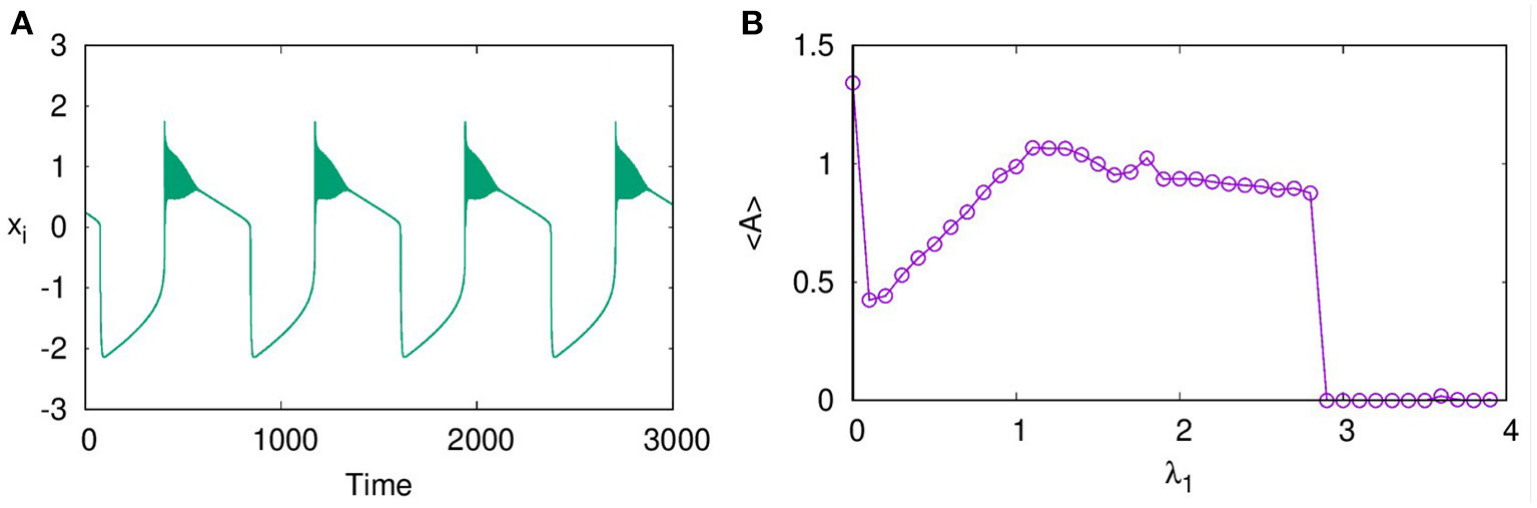
**(A)** Time series showing synchronized bursts of action potentials from all the neurons on layer L1, coupled with excitatory synaptic coupling for coupling strength λ_1_ = 1.5, **(B)** Average amplitude of oscillations for increasing coupling strength λ_1_. The transition from oscillatory state to amplitude death (AD) state occurs at λ_1_ = 2.9. Here, *p*_1_ = 1, ϵ = 0, and *N* = 50.

In layer L1 with excitatory synaptic coupling among neurons, we observe that, for sufficient strength of synaptic coupling, they settle to a completely synchronized oscillatory state, which is shown in Figure 1A at λ_1_ = 1.5. However, the nature of oscillations is changed from intrinsic bursts to varied forms like bursts of decreasing amplitudes and broad spikes as λ_1_ is increased. With stronger coupling, at λ_1_ = 2.9, these spikes are suppressed, and the layer goes to AD. We note AD phenomenon has been reported earlier in globally coupled HR neurons (Prasad et al., 2010). Here, we find that AD can occur for all values of *p*_1_, local, non-local, and global, with sufficient strength of coupling. To detect the transition to AD, we compute the average amplitude of the spikes of all the neurons using Equation (2) (Verma et al., 2017).


(2) $$<A>=(\sum_{i=1}^{N}{\langle x_{i,max}\rangle }_{t})/N$$

This is plotted in Figure 1B for *p*_1_ = 1 with increasing λ_1_. We find that the average amplitude increases with λ_1_ initially, reaches a maximum, and then decreases. At λ_1_ = 2.9, there is a sudden transition to AD. The nature of the burst patterns in these regions differs as spikes of decreasing amplitude in each burst that change to square bursts before reaching AD. We repeat the study by increasing N to 100 and 500 and find qualitatively similar results.

**Figure 2:**
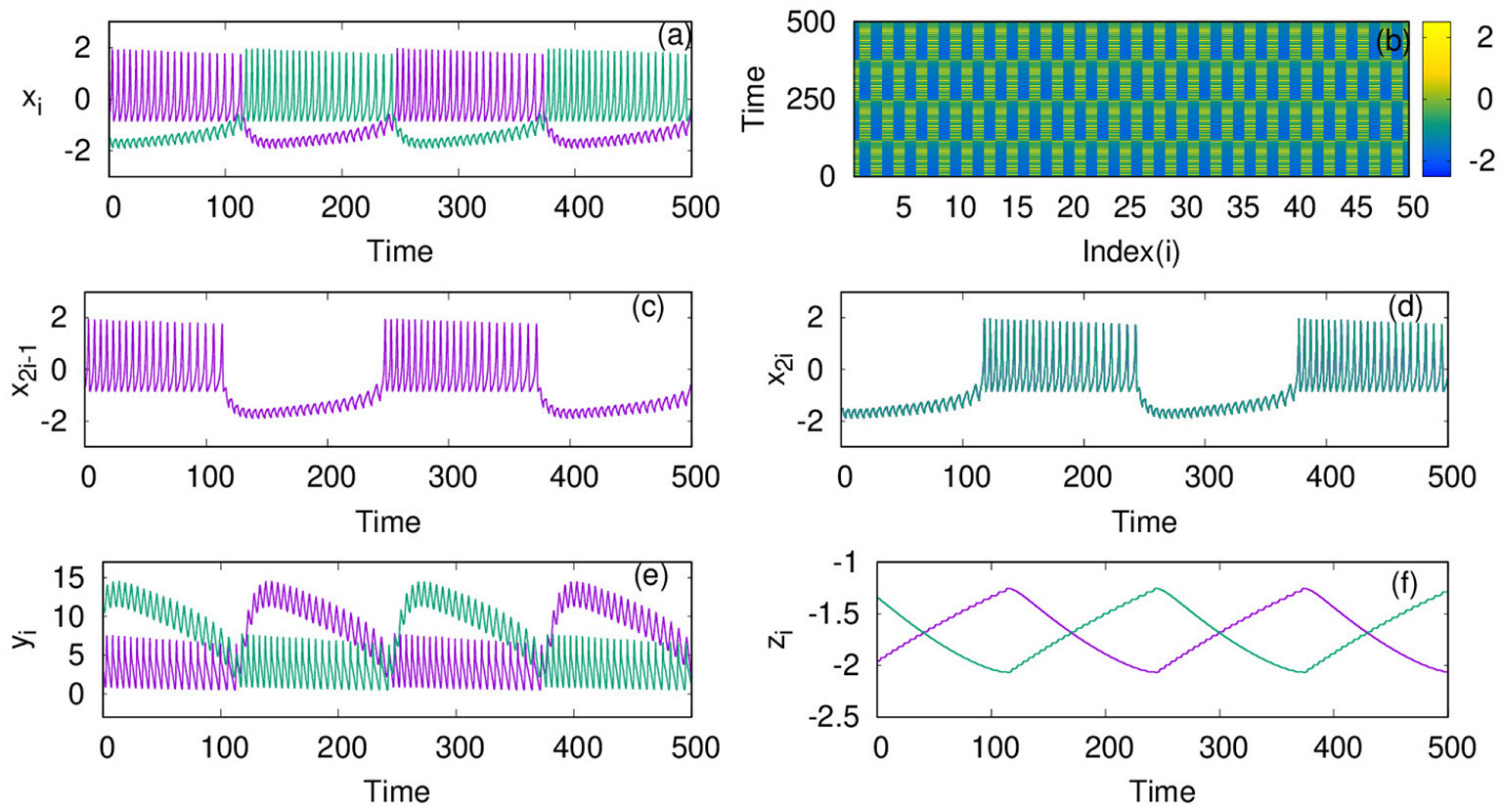
**(a)** Mixed-mode oscillations (MMO) of action potentials and **(b)** spatio temporal plot of neurons coupled with inhibitory synaptic coupling on layer L2 at coupling strength λ_2_ = 1. **(c)** Time series of all the odd number nodes *x*_2*i*−1_, showing complete synchronized oscillations, for *i* = 1;2 : : : *n*/2 and **(d)** time series of all the even number nodes *x*_2*i*_, where *i* = 1;2 : : : *n*/2 that indicate completely synchronized oscillations among them. Time series of the other variables *y*_*i*_ and *z*_*i*_ are plotted in **(e,f)**, respectively. Here, other parameters are kept as *p*_2_ = 1, ϵ = 0, and *N* = 50. The anti-phase nature of the oscillations in adjacent nodes and in-phase nature in alternate nodes separates the network into two clusters. The color bar in **(b)** (also in spatio-temporal plots in later figures) indicates the values of action potential *x*_*i*_.

For the dynamics on the second layer L2 with inhibitory synaptic coupling among neurons, we first study the case when *p*_2_ = 1, i.e., the system has only local interactions. We find that the emergent dynamics in this case shows anti-phase synchronized oscillations, which is clear from the time series, and spatio-temporal plots shown for coupling strength, λ_2_ = 1, in Figures 2a,b. First, we note that the nature of dynamics is changed from intrinsic bursts, in this case also, revealing MMO. Moreover, we find the neurons in one cluster, say at all even number sites, are all synchronized completely but are in anti-phase with those in the other cluster, at odd number sites. This is made more explicit by plotting the time series of all odd number of neurons and even number of neurons separately in Figures 2c,d, that display the pattern of anti-phase synchronized oscillations among the adjacent neurons.We also show the time series of the other two variables *y*_*i*_ and *z*_*i*_ in Figures 2e,f, respectively.

**Figure 3:**
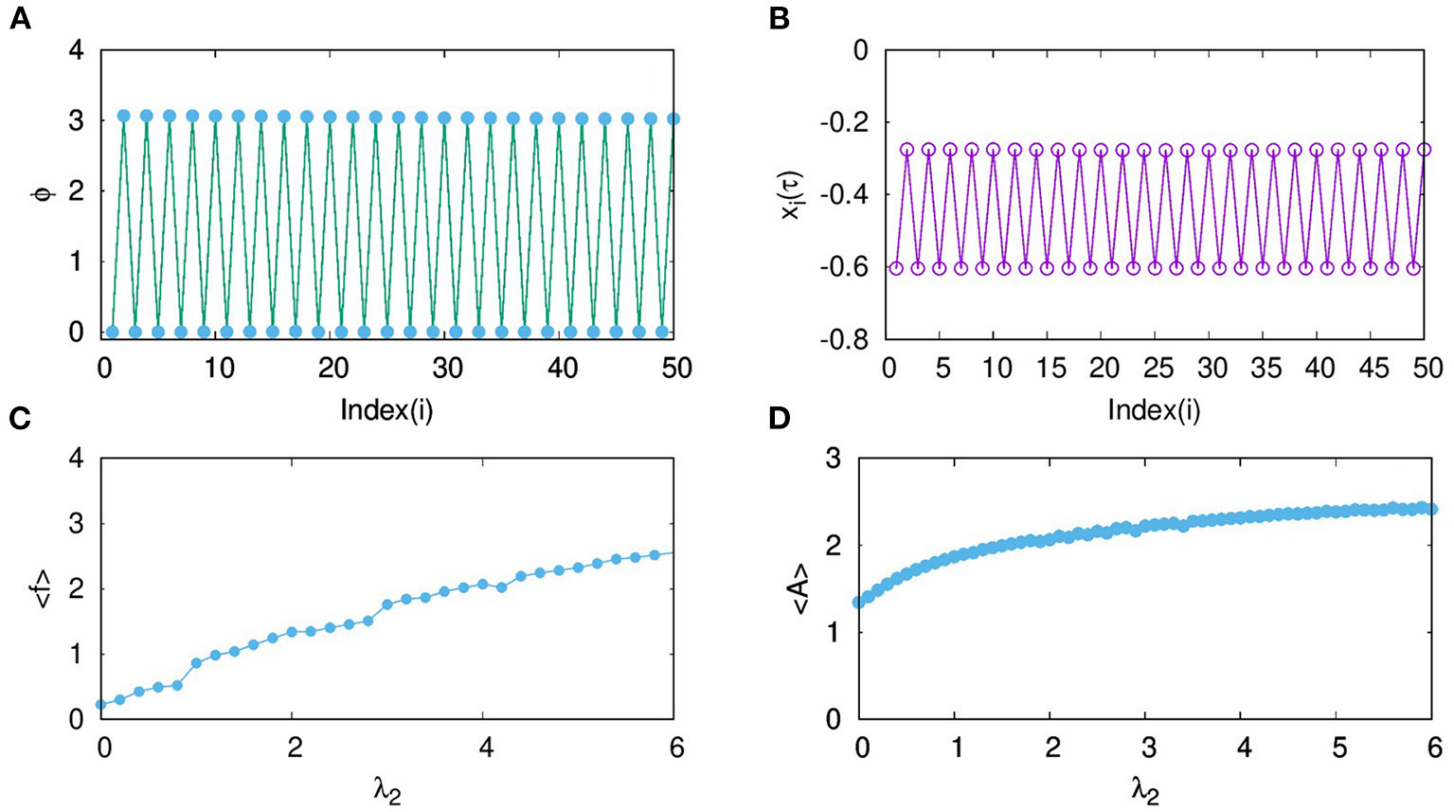
**(A)** Phase of each neuron in layer L2, coupled with inhibitory synaptic coupling calculated relative to that of its first neuron for coupling strength λ_2_ = 1 and *p*_2_ = 1. **(B)** Snapshot of *x*_*i*_ at a given time τ, shows that every odd neuron is in anti-phase with every even neuron. **(C)** Average frequency and **(D)** average amplitude of the large amplitude oscillations of the MMO for increasing coupling strength λ_2_.

For a detailed characterization of the observed phase order in temporal dynamics, we calculate the phase of each neuron from its time series, *x*_*i*_. We note the time $T_{k}^{i}$, (*k* = 1, 2, …) at which *x*_*i*_ crosses the chosen threshold value, and then, we calculate the phase of the *i*^*th*^ neuron using the following equation (Pikovsky et al., 2001):


(3) $$\phi_{i}(t)=2\pi \frac{t-T_{k}^{i}}{T_{k+1}^{i}-T_{k}^{i}},\;T_{k}^{i}\leq t\leq T_{k+1}^{i},$$

where *i* = 1, 2, …, *N*. In Figure 3A, the phase of each neuron calculated relative to that of the first neuron is plotted. It is clear that every odd neuron is in anti-phase with every even neuron. The snapshot of *x*_*i*_ at a given time τ is shown in Figure 3B, which further confirms the anti-phase pattern of the mixed mode oscillations. This is induced by the range (nearest neighbor) and the nature (inhibitory) of the coupling chosen in this context. Thus, the neurons in effect form two clusters such that synchronized oscillations in one cluster are anti-phase with that in the other cluster. Further, we calculate the spike frequency of the large amplitude oscillations of *i*^*th*^ neuron as shown in Equation (4) (Mozumdar and Ambika, 2019):


(4) $$f_{i}=\frac{2\pi }{K_{i}}\sum_{k=1}^{K_{i}}\frac{1}{t_{k+1}^{i}-t_{k}^{i}},$$

where *K*_*i*_ refers to the number of spikes for the *i*^*th*^ neuron in each burst and $t_{k}^{i}$ corresponds to time of the maximum of the *k*^*th*^ spike. Then, the average frequency obtained from this, is plotted in Figure 3C with increasing coupling strength λ_2_. Here, we can see that the average frequency increases with increasing λ_2_. We also show how the average amplitude < *A* > of coupled neurons increases with λ_2_, for the range considered as shown in Figure 3D. Both the frequency and amplitude calculated here relate to the large amplitude spikes of the mixed-mode oscillatory states of the neurons. We note such activity patterns of synchronized oscillations with amplification are reported in multiplex networks in a different context (Njougouo et al., 2020).

**Figure 4:**
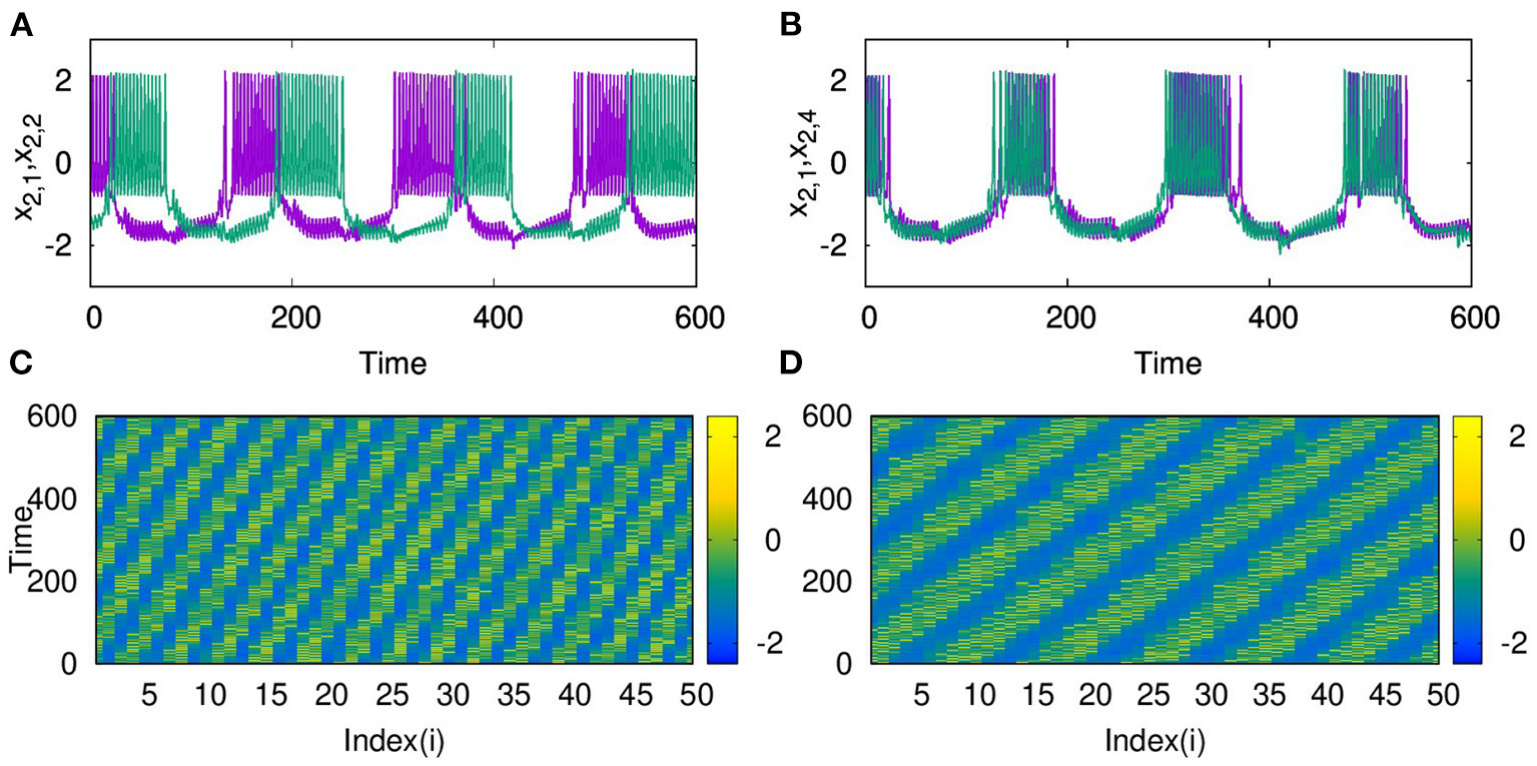
Time series of action potentials from **(A)** nodes 1 and 2 and **(B)** nodes 1 and 4 in layer L2 coupled with inhibitory synaptic coupling for *p*_2_ = 2. We find nodes *i* and *i* + 3 show inphase synchronized oscillations with small phase shift. The spatio-temporal plot of neurons showing traveling wave like patterns **(C)** for *p*_2_ = 2 **(D)** for *p*_2_ = 5. Here λ = 3 and *N* = 50.

As the range of coupling increases or the coupling becomes non-local, we observe traveling wave-like patterns. In Figure 4A, we plot the time series of the action potential from node 1 and node 2 and in Figure 4B from node 1 and node 4. It is clear that nodes 1 and 4 are almost synchronized but with a small phase shift. We find this shift in the phase depends on the coupling strength and the range of coupling. The spatio-temporal plot, in this case, shows traveling wave like patterns, as shown in Figures 4C,D, for *p*_2_ = 2 and *p*_2_ = 5, and λ_2_ = 3. For larger sizes of networks also, we find qualitatively similar emergent dynamics.

### 2.2. Dynamics of the Multiplex Network of Neurons With Excitatory and Inhibitory Synaptic Couplings

**Figure 5:**
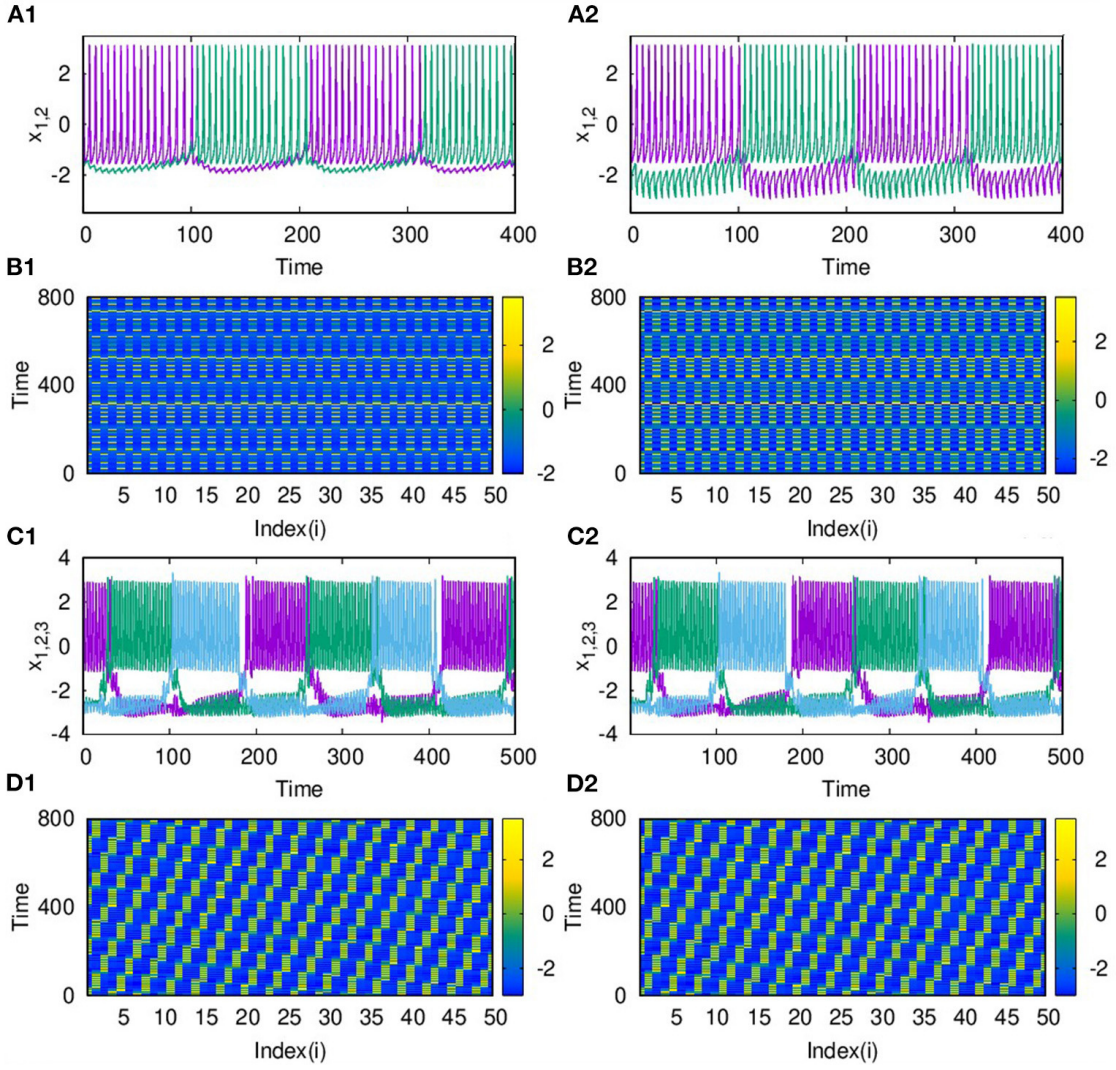
Transfer of dynamical patterns from L2 to L1, in the 2-layer multiplex network with neurons on L2 coupled with inhibitory synaptic coupling and neurons in L1 uncoupled. Time Series of action potential and spatio-temporal plot are shown for different values of *p*_2_ and λ_2_: **(A1,B1)** first layer and **(A2,B2)** second layer with *p*_2_ = 1 and λ_2_ = 6. **(C1,D1)** first layer and **(C2,D2)** second layer, with *p*_2_ = 2 and λ_2_ = 10. Here λ_1_ = 0, ϵ = 2, and *N* = 50.

With the two layers of neurons multiplexed, we study how different emergent activity patterns of dynamics get selected across the layers as parameters are varied. We first consider the case where neurons of layer L1 are uncoupled, while those of layer L2 are coupled with inhibitory synaptic coupling and both layers are coupled to each other via *i* to *i* connections with feedback coupling of strength ϵ as given in Equation (1). In this case, with *p*_2_ = 1, λ_2_ = 6 for L2, ϵ = 1, the patterns of synchronized oscillations that are anti-phase for adjacent nodes on second layer L2, get selected as such in the first layer L1 also. This is clear from Figures 5A1–B2, where the time series and spatio-temporal plots of both layers are given. Also for *p*_2_ = 2 and λ_2_ = 10, both layers show traveling wave-like oscillations (Figures 5C1–D2). Thus, the emergent dynamics and the corresponding activity patterns get transferred from one layer to other layer when the layers are multiplexed.

**Figure 6:**
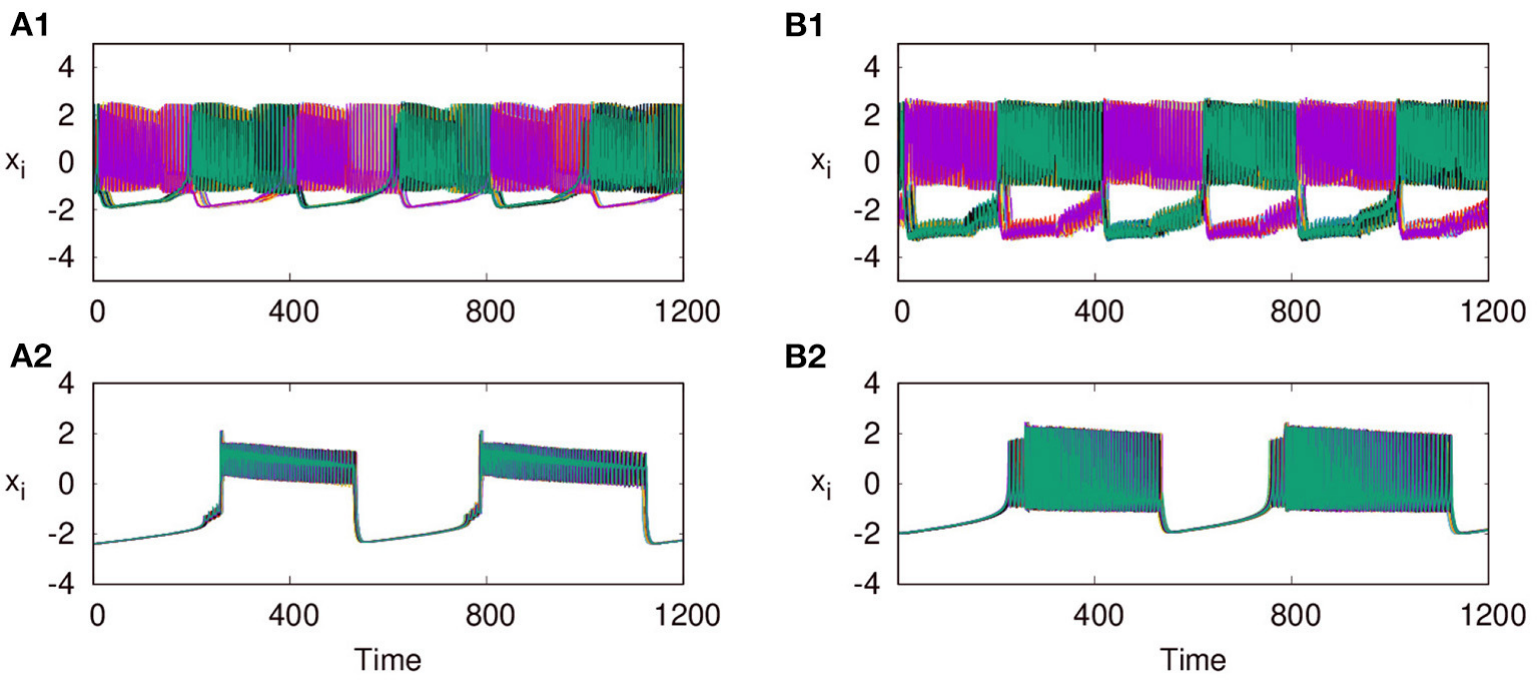
Time series of the action potentials of multiplex HR neurons for both layers L1 (left panel) and L2 (right panel) for different values of λ_1_ and λ_2_: **(A1,B1)** λ_1_ = 0.1 and λ_2_ = 1.0, show anti-phase synchronized oscillations in two clusters, and **(A2,B2)** λ_1_ = 3 and λ_2_ = 0.1, show in-phase synchronized oscillations. Here ϵ = 1, *p*_1_ = 1, *p*_2_ = 1, and *N* = 50. The pattern of the dynamics on the layer of larger intralayer coupling strength is selected across both layers.

Next, we consider the neurons of first layer L1 coupled with excitatory synaptic coupling, with neurons of L2 still coupled with inhibitory synaptic coupling, both with local couplings as *p*_1_ = 1, and *p*_2_ = 1. With the interlayer coupling strength at ϵ = 1, for λ_1_ = 0.1, and λ_2_ = 4, we observe that both layers L1 and L2 exhibit anti-phase synchronized oscillations, with phase ordering which is shown in Figures 6A1,B1, respectively. When we set λ_1_ = 3.0 and λ_2_ = 0.1, we observe in-phase synchronized oscillations in both layers, which is shown in Figures 6A2,B2, respectively. Thus, we see that for strong inhibitory synaptic coupling strength, both layers show anti-phase synchronized oscillations in adjacent nodes, while for strong excitatory synaptic coupling, both layers show in-phase synchronized oscillations.

**Figure 7:**
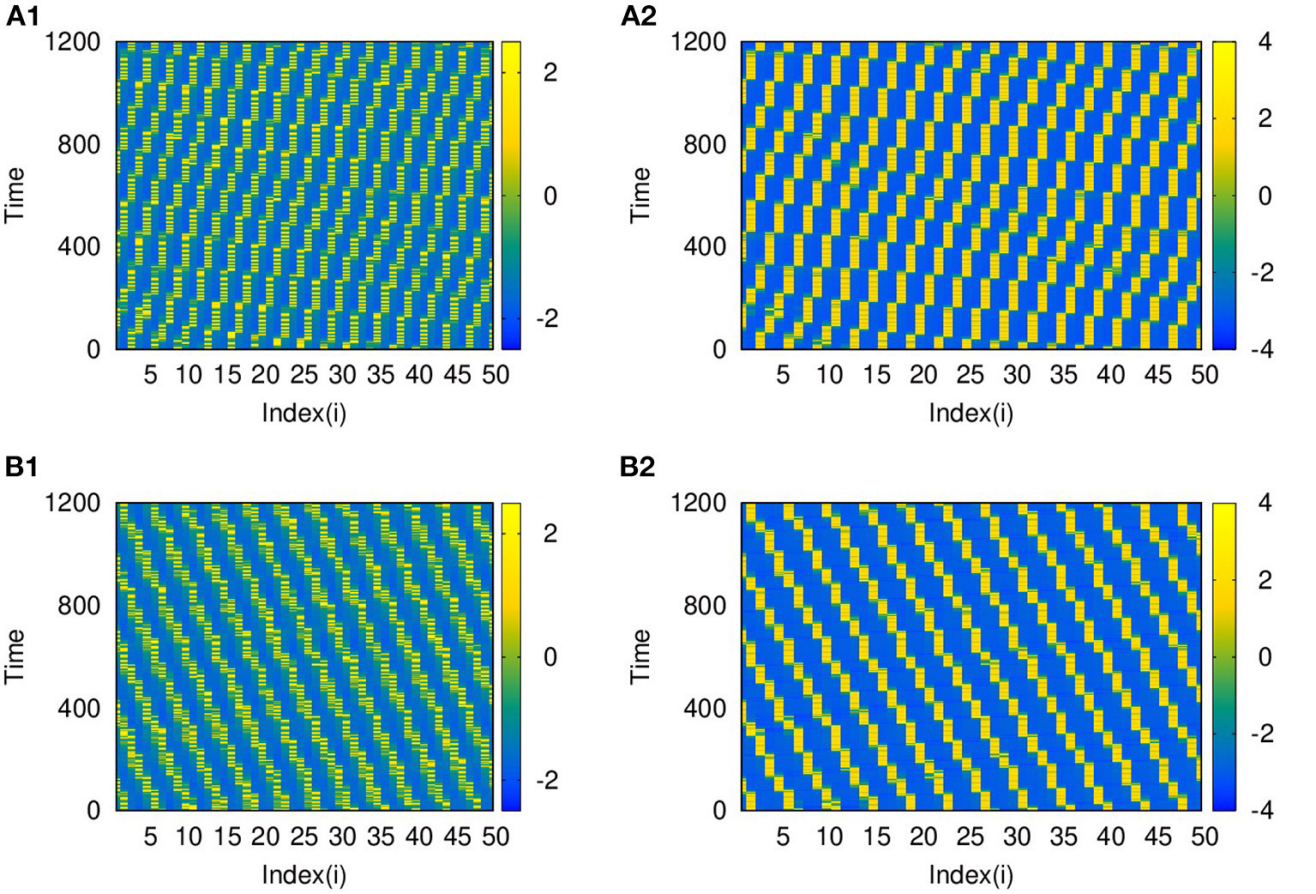
Spatio-temporal plots of multiplex HR neurons for layer L1 (left panel) and L2 (right panel) for different values of *p*_2_, andλ_2_: **(A1,B1)** *p*_2_ = 2 and λ_2_ = 6, **(A2,B2)** *p*_2_ = 3, and λ_2_ = 10. Here, λ_1_ = 0.1, ϵ = 1, *p*_1_ = 10, and *N* = 50. The spatio-temporal dynamics of the layer with larger coupling strength gets selected in both the layers. However, the nature of spikes and bursts are different in L1 and L2 due to difference in intralayer parameters.

Also, as couplings become non-local, with *p*_2_ = 2 and 3, both layers show phase shifted oscillations and spatio-temporal dynamics that are transferred from L2 to L1 for larger λ_2_. We also observe that these states are selected by layer 1 for all values of *p*_1_ up to *p*_1_ = 10, λ_1_ = 0.1. The spatio-temporal plots for *p*_2_ = 2, and λ_2_ = 6, shown in Figures 7A1,B1, and for *p*_2_ = 3 and λ_2_ = 10, in Figures 7A2,B2, indicate the transfer of dynamical patterns across the layers. However, the nature of spikes and bursts in layers L1 and L2 differs due to difference in the parameter chosen. So, the selection of the specific activity patterns on both layers depends on the relative intra-layer coupling strengths and follows the spatio-temporal dynamics of the layer with larger intra-layer coupling strength. This is further illustrated for other types of emergent dynamics below.

**Figure 8:**
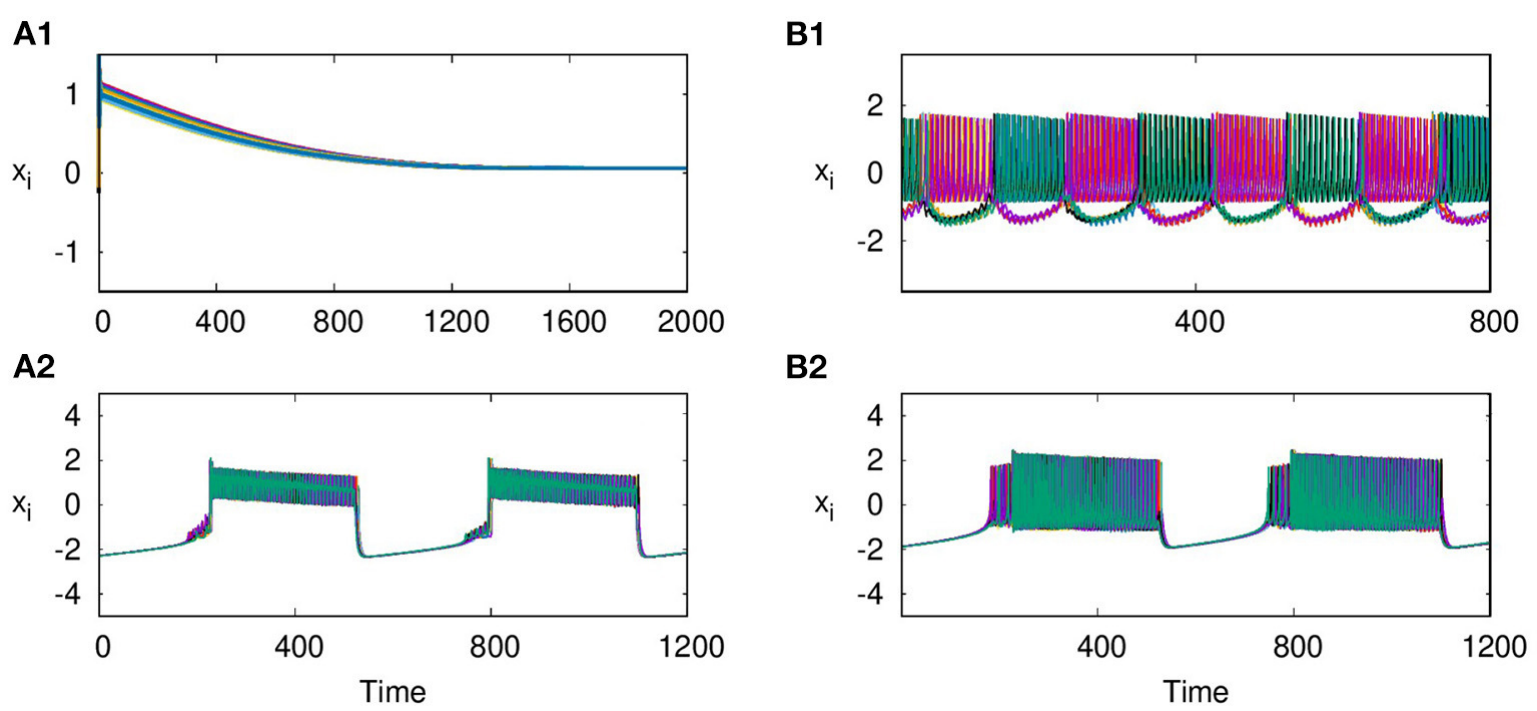
Revival of activity in layer L1 due to multiplexing with L2 and transition from in phase to anti-phase in L2 as parameters are tuned. Time series of action potentials for multiplex HR neurons in layer L1 (left panel) and L2 (right panel) are plotted for different values of λ_1_ and λ_2_: **(A1)** at λ_1_ = 3, ϵ = 0 neurons in layer L1 exhibit AD, **(B1)** at λ_2_ = 0.3, and ϵ = 0: neurons in layer L2 show anti-phase oscillations in two clusters: **(A2,B2)** λ_2_ = 0.3 and ϵ = 1: observed revival of oscillations in L1 and transition from anti-phase to in-phase in L2. Here, λ_1_ = 3, *p*_1_ = 1, *p*_2_ = 1, and *N* = 50.

**Figure 9:**
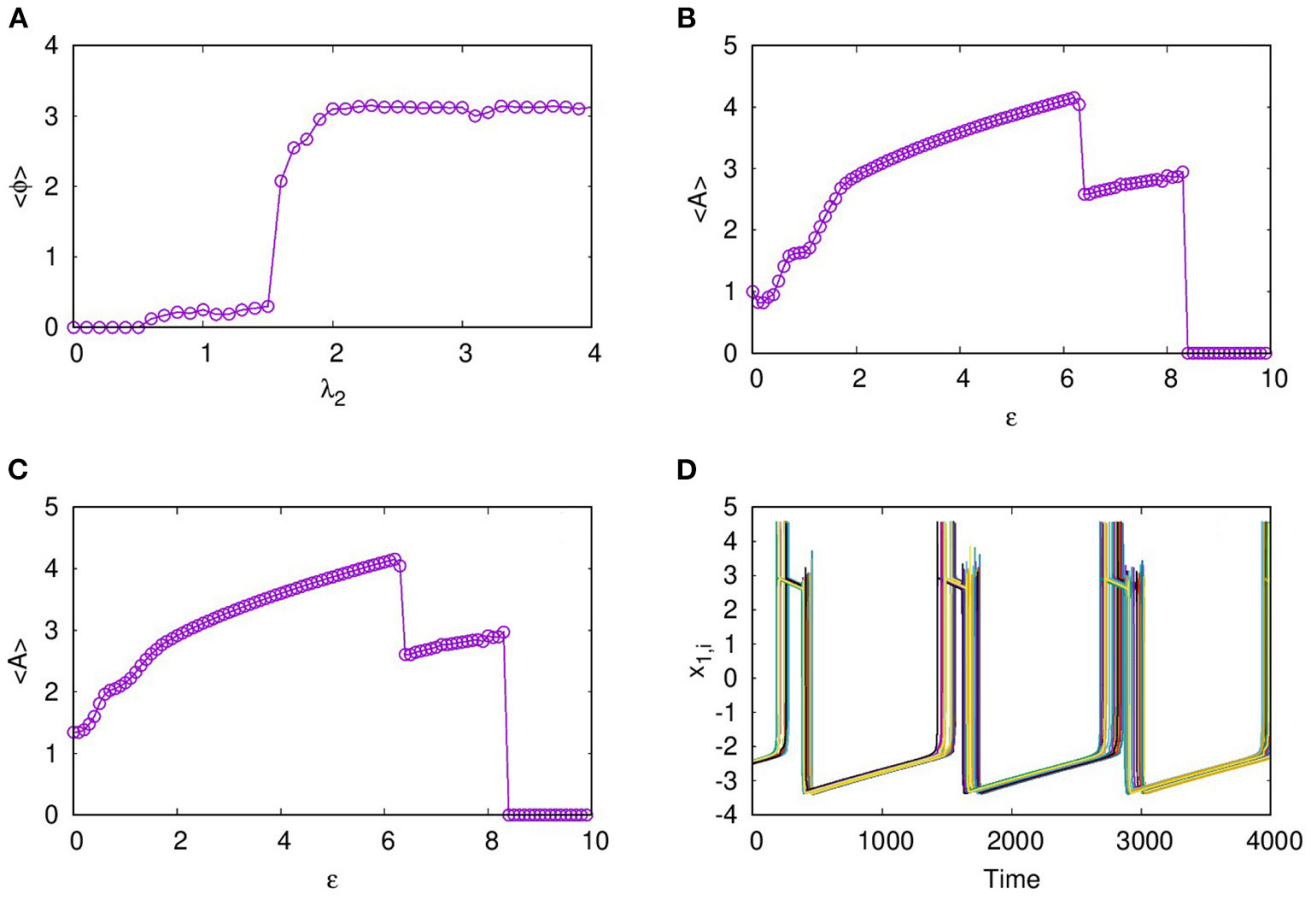
**(A)** Transition from in-phase to anti-phase for synchronized activity in L2. Here, average phase of neurons on the second layer for varying coupling strength λ_2_, is shown with neurons in L1 coupled locally with *p*_1_ = 1 and λ_1_ = 3. Suppression of activity on increasing the interlayer coupling strength. Average amplitudes of neurons on **(B)** L1 and **(C)** L2 are shown with varying interlayer coupling strength ϵ. The nature of spikes and bursts near the transition for ϵ = 8.1 is shown in **(D)**. Here, λ_1_ = 1, λ_2_ = 1.0, *p*_1_ = 1, *p*_2_ = 1, and *N* = 50.

As reported earlier, when ϵ = 0, both L1 and L2 function as independent layers, and for higher synaptic coupling strength λ_1_ = 3, layer L1 goes to AD and at λ_2_ = 0.3, layer L2 shows anti-phase synchronized oscillations in two clusters (Figures 8A1,B1). But when both layers are multiplexed with ϵ = 1, we observe a revival of oscillations from death state on layer L1 and in-phase oscillation on layer L2, as shown in Figures 8A2,B2, respectively. Further, we observe that the activity pattern in L2 undergoes a transition from in-phase to anti-phase as λ_2_ is tuned. This transition from in-phase to anti-phase with an increase in λ_2_ in layer L2 is shown in Figure 9A, where the average phase difference is calculated as $<\phi >=\frac{1}{N}\overset{N}{\underset{i=1}{\sum }}\left(\phi_{i}-\phi_{i+1}\right)$, with ϕ_*i*_ obtained for each neuron from Equation (3). The inhibitory synaptic coupling in one layer can revive the oscillations from the suppressed state on the other layer. The variety of interesting activity patterns of spatio-temporal dynamics and their selection across layers happens at low to moderate values of interlayer coupling strengths. When the interlayer coupling strength ϵ is increased, to say ϵ = 10, both layers settle to AD states (Figures 9B,C), and the time series near the transition point is as shown in Figure 9D. Thus, the selection of activity patterns in both layers due to multiplexing depends on the nature and strengths of intralayer and interlayer couplings, and therefore, the coupling strengths and range of couplings can be tuned to select any desired pattern of activity.

### 2.3. Dynamics of the Multiplex Network of Neurons With Electrical and Synaptic Coupling

Now, we consider the case where neurons in the first layer (L1) interact with each other with electrical coupling and those in the second layer (L2) interact through synaptic coupling. The dynamics of the multiplex network of neurons thus modeled is given as follows,


(5) $$\begin{array}{l} {\dot{x}}_{i,1}=B_{i,1}+\frac{\lambda_{1}}{2p_{1}}\sum_{k=i-p_{1}}^{i+p_{1}}\left(x_{k,1}-x_{i,1}\right)+\epsilon x_{i,2}\\ {\dot{y}}_{i,1}=(a+\alpha )x_{i,1}^{2}-y_{i,1}\\ {\dot{z}}_{i,1}=c(bx_{i,1}-z_{i,1}+e)\\ {\dot{x}}_{i,2}=B_{i,2}+E\frac{\lambda_{2}}{2p_{2}}(V_{s}-x_{i,2})\sum_{k=i-p_{2}}^{i+p_{2}}\Gamma (x_{k,2})+\epsilon x_{i,1}\\ {\dot{y}}_{i,2}=(a+\alpha )x_{i,2}^{2}-y_{i,2}\\ {\dot{z}}_{i,2}=c(bx_{i,2}-z_{i,2}+e), \end{array}$$

Here, we define a parameter E whose sign decides the nature of synaptic coupling, for *E* = 1 neurons in L2 are coupled with excitatory synaptic coupling, and for *E* = −1, second layer are coupled with inhibitory synaptic coupling.

**Figure 10:**
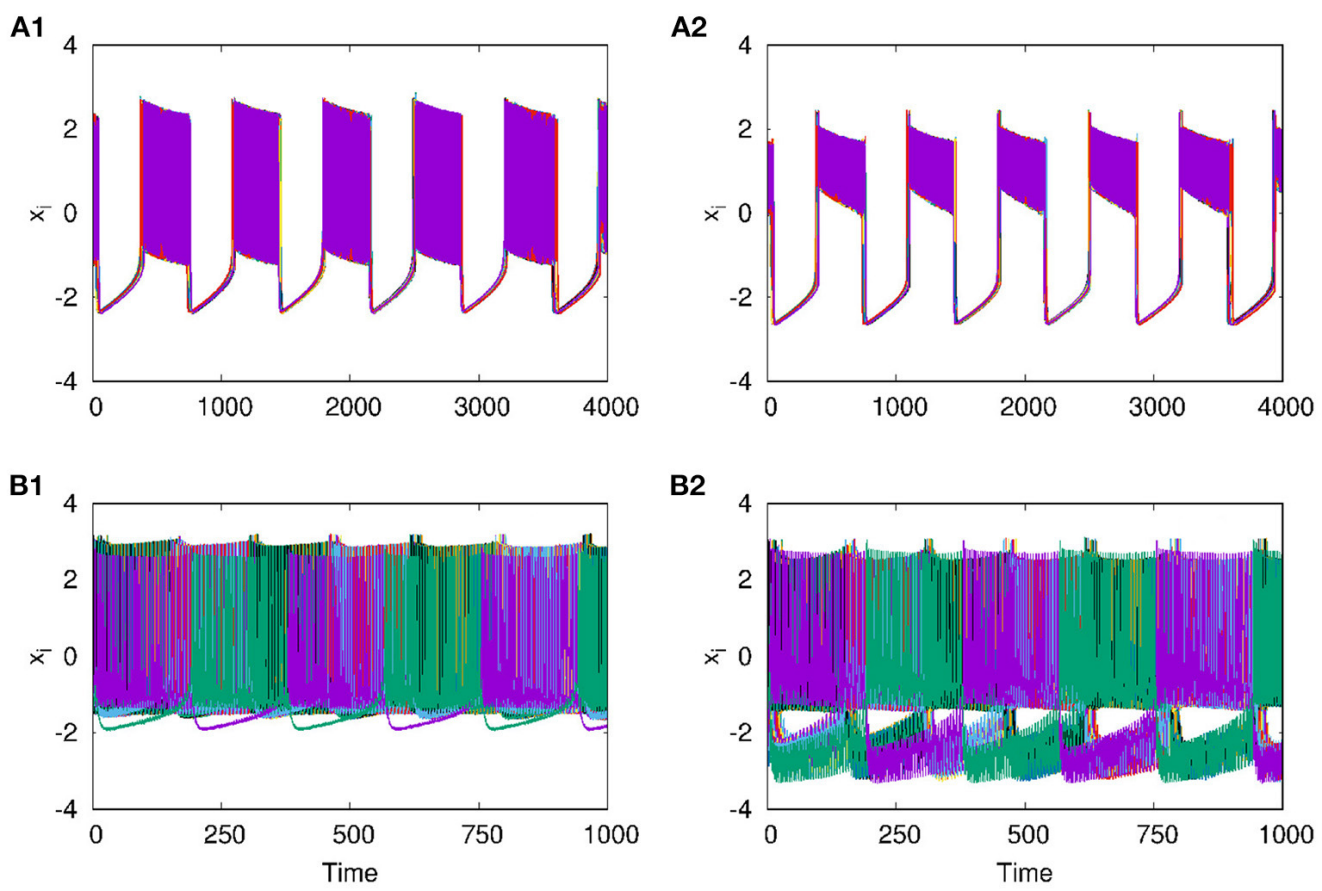
Time series of action potentials for multiplex HR neurons from layer L1 (left panel) to L2 (right panel): **(A1,B1)** in-phase patterns for neurons of L1 coupled with electrical coupling and that of L2 coupled with excitatory synaptic coupling. **(A2,B2)** Anti-phase activity for neurons on L1 coupled with electrical coupling and that on L2 coupled with inhibitory synaptic coupling. The dynamics of L2 are transferred to L1 in both cases. λ_1_ = 0.5, λ_2_ = 5, *p*_1_ = 1, *p*_2_ = 1, and *N* = 50.

**Figure 11:**
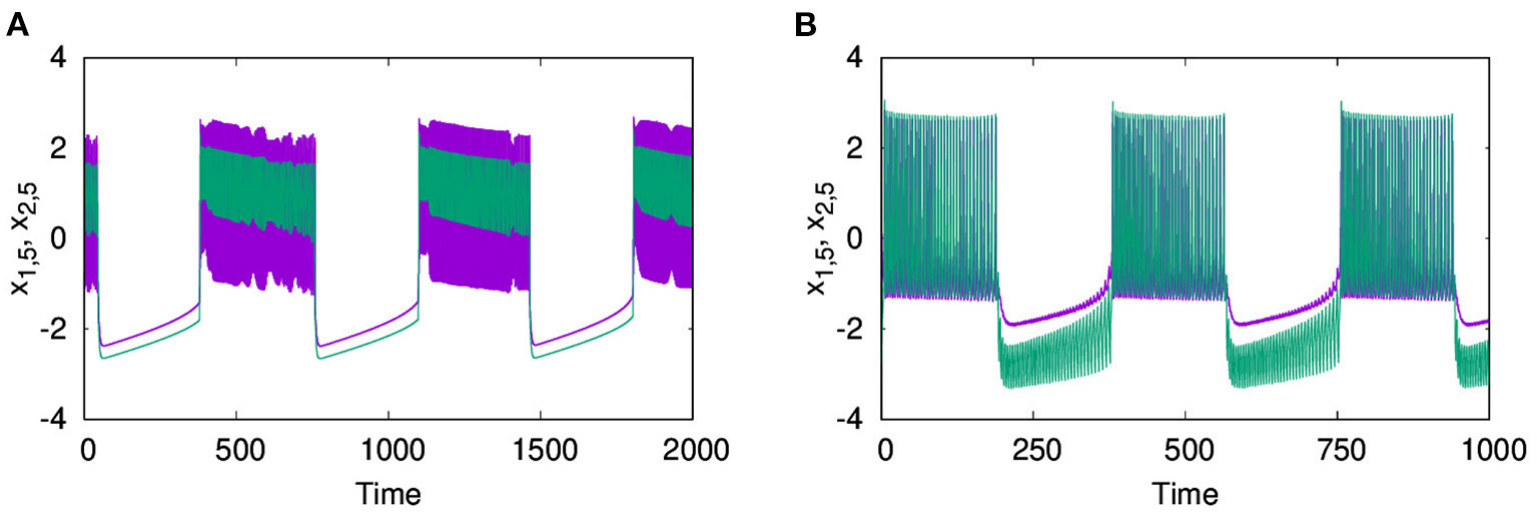
In-phase synchronized dynamics between similar nodes on layers L1 and L2. Time series of node 5 from both layers L1 and L2 are plotted with **(A)** neurons of L1 coupled with electrical coupling and that of L2 coupled with excitatory synaptic coupling; **(B)** neurons of L1 coupled with electrical coupling and that of L2 coupled with inhibitory synaptic coupling. The other parameters are λ_1_ = 0.5, λ_2_ = 5, *p*_1_ = 1, *p*_2_ = 1, and *N* = 50.

With *E* = 1, and the excitatory coupling strength at λ_2_ = 5, we observe that the coupled system shows in-phase synchronized oscillations, in both layers L1 and L2, as shown in Figures 10A1,B1, respectively. Next, with *E* = −1, the coupled system shows anti-phase synchronized oscillations in both layers L1 and L2 (Figures 10A2,B2). Further, we also observe that along with the transfer of the emergent phenomena from one layer to another, the node of layer L1 shows in-phase synchronization with the same node of layer L2. To indicate this, we show the time series of the 5^*th*^ node of both layers L1 and L2, where layer L1 coupled with electrical coupling and L2 coupled with excitatory coupling in Figure 11A and L2 coupled inhibitory synaptic coupling in Figure 11B, respectively. Also, for strong electrical coupling strength (λ_1_) and weak synaptic coupling (λ_2_) (inhibitory or excitatory), we observe traveling wave patterns on both layers.

## 3. Conclusion

In this study, we report the selection of various activity patterns as the emergent spatio-temporal dynamics on a multiplex neuronal network of HR neurons where the nature of interaction in each layer can be different. This framework can thus model the plasticity and variability of connections among neurons which can exist as synaptic or electrical in nature with excitatory or inhibitory connections. By tuning the strengths of connections in each layer and across layers, the network can select various activity patterns and induce the pattern from one layer to the other.

We first present the pattern of dynamics on the first layer L1, where neurons are coupled through excitatory synaptic couplings. By tuning the synaptic coupling strength, the coupled neurons can be in completely synchronized oscillations, while for strong synaptic coupling strength, the oscillations are suppressed to the state of AD. The phenomenon of AD is observed for all values of *p*_1_, corresponding to the local, non-local, and global types of couplings. The second layer of neurons, coupled with inhibitory synaptic coupling, shows anti-phase synchronized oscillations with amplification when the neurons are locally coupled, i.e., *p*_2_ = 1. The anti-phase synchronized oscillations are interesting in two aspects. First, the nature of oscillations are MMO with enhanced frequency and amplitude with large amplitude spikes, and second, the phase relationship among them occurs in an orderly way, with alternate neurons being in phase and neighboring ones being in anti-phase. Thus, the whole network splits into two clusters, every odd node belonging to one cluster and every even node to the other cluster. For *p*_2_ = 2 and 3, we get traveling wave type of oscillations over the network.

When the two layers are multiplexed, for sufficient inhibitory coupling strength, we observe mixed-mode synchronized oscillations that are phase-shifted get selected on both layers. In general, the selection of the specific pattern of activity on both layers can be controlled by tuning the relative intra-layer coupling strengths.

Also, multiplexing can revive the oscillations from the AD state on the first layer by changing the inhibitory coupling strength on the second layer. We also report the transition from anti-phase to the in-phase type of MMO, and vice versa that get selected as the excitatory and inhibitory coupling strengths are tuned to specific values. We repeat the study by increasing the size of the networks in both layers to 100 and 500 and find qualitatively similar results.

With the nature of coupling among neurons in one layer L1 electrical, while the other layer L2 has neurons with synaptic connections, we observe in-phase synchronized activity in both layers when L2 has excitatory connections and anti-phase activity when it has inhibitory connections. We also find neurons at similar nodes in both layers are synchronous with in-phase oscillations.

We note the variety of activity patterns presented here that occur for a collection of neurons forming a multiplex network, corresponding to experimentally observed patterns of activity reported recently (Crofts et al., 2016). Also, modulation of neuronal oscillation frequency is reported to occur during sensory information processing (Lee et al., 2018). Thus, the study provides a better understanding of the mechanism underlying such patterns known to occur in brain networks that incorporate multiplex network architecture naturally (Frolov et al., 2020).

## Data Availability Statement

The raw data supporting the conclusions of this article will be made available by the authors, without undue reservation.

## Author Contributions

GA and UK conceived and designed the study. UK performed the numerical analysis and drafted the manuscript. All authors edited and approved the manuscript.

## Conflict of Interest

The authors declare that the research was conducted in the absence of any commercial or financial relationships that could be construed as a potential conflict of interest.

## Publisher's Note

All claims expressed in this article are solely those of the authors and do not necessarily represent those of their affiliated organizations, or those of the publisher, the editors and the reviewers. Any product that may be evaluated in this article, or claim that may be made by its manufacturer, is not guaranteed or endorsed by the publisher.
